# Clinical application and detection techniques of liquid biopsy in gastric cancer

**DOI:** 10.1186/s12943-023-01715-z

**Published:** 2023-01-11

**Authors:** Shuo Ma, Meiling Zhou, Yanhua Xu, Xinliang Gu, Mingyuan Zou, Gulinaizhaer Abudushalamu, Yuming Yao, Xiaobo Fan, Guoqiu Wu

**Affiliations:** 1 Center of Clinical Laboratory Medicine, Zhongda Hospital, Southeast University, Nanjing, 210009 Jiangsu China; 2 Department of Laboratory Medicine, Medical School of Southeast University, Nanjing, 210009 Jiangsu China; 3 Department of Laboratory Medicine, Northern Jiangsu People’s Hospital Affiliated to Yangzhou University, Yangzhou, 225000 Jiangsu China; 4 Department of Laboratory Medicine, Medical School, Affiliated Hospital of Nantong University, Nantong University, Nantong, 226001 Jiangsu China; 5 Jiangsu Provincial Key Laboratory of Critical Care Medicine, Southeast University, Nanjing, 210009 Jiangsu China

**Keywords:** Gastric cancer, Liquid biopsy, Circulating tumor cells, Circulating tumor DNA, Non-coding RNAs, Exosomes

## Abstract

Gastric cancer (GC) is one of the most common tumors worldwide and the leading cause of tumor-related mortality. Endoscopy and serological tumor marker testing are currently the main methods of GC screening, and treatment relies on surgical resection or chemotherapy. However, traditional examination and treatment methods are more harmful to patients and less sensitive and accurate. A minimally invasive method to respond to GC early screening, prognosis monitoring, treatment efficacy, and drug resistance situations is urgently needed. As a result, liquid biopsy techniques have received much attention in the clinical application of GC. The non-invasive liquid biopsy technique requires fewer samples, is reproducible, and can guide individualized patient treatment by monitoring patients' molecular-level changes in real-time. In this review, we introduced the clinical applications of circulating tumor cells, circulating free DNA, circulating tumor DNA, non-coding RNAs, exosomes, and proteins, which are the primary markers in liquid biopsy technology in GC. We also discuss the current limitations and future trends of liquid biopsy technology as applied to early clinical biopsy technology.

## Introduction

Gastric cancer (GC) is the fifth most common type of cancer and the third leading cause of death worldwide [1]. The morbidity and mortality rates of GC are increasing because most GC patients are already at an advanced stage of cancer when diagnosed [2]. Because GC has a poor prognosis, few treatment options, and is prone to metastasis, recurrence, and drug resistance [3], a reliable tool for early GC screening and predicting treatment efficacy is required. Endoscopy, *Helicobacter pylori* serology, and serum pepsinogen testing are the most common clinical methods for GC screening [4]. Surgical resection, chemotherapy, and targeted therapy are the primary treatment modalities for GC [5]. Endoscopic tissue biopsy, the gold standard for GC screening, is a relatively expensive and invasive procedure with varying degrees of patient harm; a single biopsy does not typically reflect the heterogeneity of GC patients, and the sensitivity and specificity of this method are low due to tissue resection site limitations [6–8]. Furthermore, carcinoembryonic antigen (CEA), carbohydrate antigen (CA) 199, CA724, CA125, CA242, pepsinogen, and alpha-fetoprotein are extensively used clinical markers for early GC screening. However, their specificity and sensitivity are low and lack GC-specific [9]. Due to the limitations of these screening methods, there is an urgent need to develop a minimally invasive method for early detection and therapeutic decision-making in GC. Therefore, a new promising screening modality, liquid biopsy, has been investigated and validated for clinical use in GC patients.

**Fig. 1:**
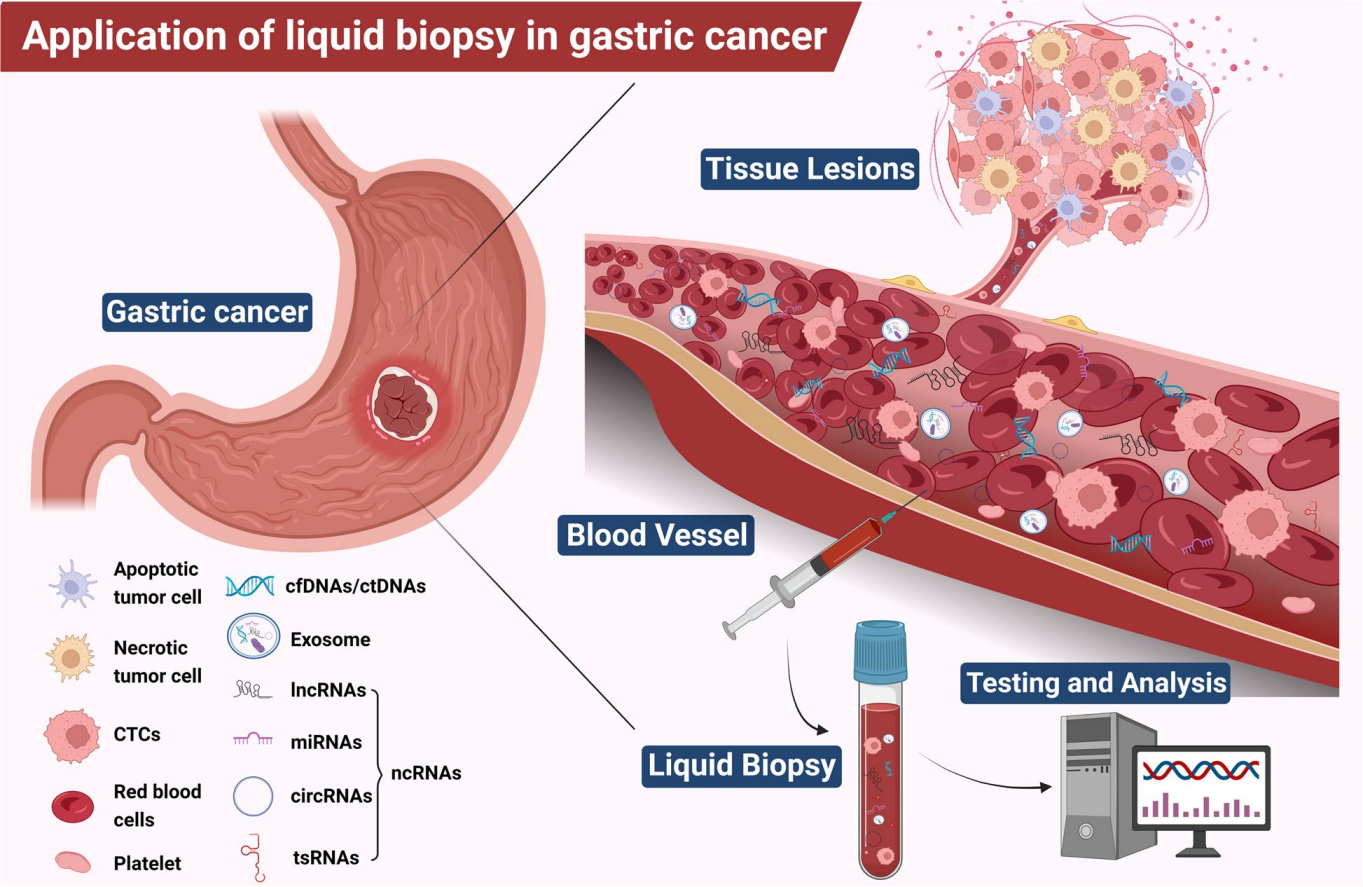
Clinical application of liquid biopsy in gastric cancer (GC). Circulating tumor cells (CTCs), circulating free DNA (cfDNA), circulating tumor DNA (ctDNA), non-coding RNAs (ncRNAs), exosomes, and proteins in the blood of GC patients can be used as potential biomarkers for liquid biopsies and their expression levels can be measured to reflect the clinical status of GC. Created with BioRender.com

Liquid biopsy is a non-invasive technique for detecting and analyzing circulating tumor cells (CTCs), circulating free DNA (cfDNA), circulating tumor DNA (ctDNA), non-coding RNAs (ncRNAs), exosomes, and proteins in biological fluid samples (such as blood, saliva, pleural fluid, ascites, stool, urine, and cerebrospinal fluid) [10, 11]. The possible sources of liquid biopsy are illustrated in Fig. 1. Liquid biopsies have many advantages over traditional tissue biopsies. Liquid biopsies, for example, require fewer samples and can be repeated [12]. Liquid biopsy technology can be used to investigate tumor load and genetic changes in patients throughout their disease by monitoring changes at the molecular level in real-time, and it can also be used to make decisions and adjustments to subsequent treatment options [13]. Furthermore, because of the minimally invasive nature of liquid biopsy technology, it has promising clinical applications for early diagnosis and screening of GC patients, prognostic monitoring, early recurrence detection, and longitudinal monitoring of disease progression and treatment response during adjuvant and neoadjuvant therapy [14, 15]. Studies show that liquid biopsy techniques can identify GC patients in novel ways.

This review focuses on the clinical applications of liquid biopsy technology’s primary markers for early diagnosis, prognosis prediction, recurrence, metastasis monitoring, chemotherapy sensitivity, and drug resistance in GC. Furthermore, we briefly describe the main liquid biopsy techniques for detecting different biomarkers in GC and summarize their clinical utility for GC patients. Finally, we discuss the limitations of liquid biopsy techniques in GC biology and speculate on their future development, which opens new avenues for GC clinical applications.

## Clinical application of liquid biopsy biomarkers in GC

### CTCs

In 1869, Thomas Ashworth discovered CTCs, a type of tumor cell shed into the bloodstream from primary or metastatic tumor sites [16]. CTCs are circulating nucleated cells with a diameter larger than 4 μM that can express epithelial cell adhesion molecules (EpCAM), cytokeratins (CKs) 8, 18, and/or 19, and do not use CD45 as a rejection marker for leukocytes, which are the primary basis for CTC detection [17]. CTCs are highly heterogeneous, allowing them to easily evade immune surveillance and treatment, eventually resulting in distal metastasis or tumor cell recurrence [18–20]. Furthermore, due to CTCs’ short half-life (about 1–2.4 h) [21], the level of CTCs in the blood is low [22]. Despite the numerous challenges in detecting CTCs in GC, new techniques can still detect the type and number of CTCs and thus determine tumor progression [23, 24]. Liquid biopsy of CTCs has been used for decades to aid in early diagnosis, prognostic stratification, dynamic assessment, and guide treatment decisions for patients with GC [25, 26]. The clinical application of CTCs as liquid biopsy markers in GC is listed in Table 1.

**Table 1:** Clinical application of CTCs in GC

Study type	Threshold	Sample size	Sensitivity (%)	Specificity (%)	AUC	Clinical significance	References
Retrospective study	2 CTCs	116 patients with GC	85.3	90.3	0.928	Distinguish between GC patients and healthy controls and provide clinical thresholds	[27]
	Number of CTCs	20 studies	42	99	0.97	Differentiate between GC patients and healthy controls	[28]
	Single CTCs	24 patients with metastatic GC receiving chemotherapy	96	^a^	^a^	Detection of metastasis and drug resistance in GC	[29]
	CTCs/DTCs	26 studies	^a^	^a^	^a^	As the basis for GC staging	[30]
	CSV + PD-L1 + CTCs	70 patients with GC	71	^a^	^a^	Predicts treatment response and prognosis in GC patients	[31]
	CTC-PD-L1	32 patients with progressive GC	^a^	^a^	^a^	Monitor prognosis and guide future individualized immunotherapy	[32]
Prospective study	CTCs and TWIST	32 patients with metastatic GC	80.6	^a^	^a^	As a prognostic marker	[33]
	4 CTCs	52 patients with progressive GC	^a^	^a^	^a^	As a surrogate marker for the efficacy of treatment with S-1 or paclitaxel in AGC patients	[34]
	5 CTCs	65 treatment^a^negative gastric adenocarcinomas	^a^	^a^	^a^	Monitoring the prognosis and recurrence of GC	[35]
	2 CTCs	44 patients with gastrointestinal tumors	69.9	^a^	^a^	Determining the prognosis of metastatic GC	[36]
	FR + CTCs	132 patients with GC	77.8	54.5	0.68	Preoperative testing of FR + CTC levels helps predict PM and early recurrence in GC patients	[37]
	CTCs/cfDNA	45 patients with progressive GC	95.6	^a^	^a^	Predicting the efficacy and prognosis of neoadjuvant chemotherapy for progressive GC	[38]

^a^indicates that this data was not presented in the study

#### Clinical application of CTCs in GC liquid biopsy

Because 80% of GC patients are asymptomatic in the early stages, early screening of patients is one of the most important challenges in GC [39]. In recent years, CTCs’ role in diagnosing GC has received increased attention. Kang et al. [27] detected CTCs in 90.5% (105/116) of GC patients and identified GC patients with a CTC ≥ 2/7.5 mL of blood threshold. Their sensitivity and specificity in distinguishing GC patients from healthy controls are 85.3% and 90.3%, respectively. Their conclusion points out that although CTCs were not associated with the T or N stages, the detection rate of CTCs in patients with T1 and N0 stages GC was more than 80%. Similarly, Tang et al. [28] found that the sensitivity of using CTCs to detect patients with advanced GC was higher than that of detecting patients with early GC, but the specificity was almost the same. Because of the low sensitivity of the detection, they suggested that CTCs could not be used for separate screening of GC, which also suggested that CTCs should be combined with markers with higher sensitivity for better results.

In GC, CTCs have been linked to metastasis, prognosis, recurrence, and chemotherapy [40]. Hiraiwa et al., the first to investigate the clinical significance of CTCs in GC patients using the CellSearch system, found that the detection rate of two CTCs was 69.9%, two or more CTCs were significantly related to advanced tumor stage in GC patients, and the patients with distant organ metastases from GC have significantly higher numbers of CTCs than healthy controls and non-metastatic patients [36]. Similarly, Jhi et al. [33] and Negishi et al. [29] found CTCs in 80.6% (25/31) and 96% (26/27) of patients with metastatic GC, respectively, and the number of CTCs in the blood correlates with overall survival (OS). The above findings are also consistent with Ito et al.’s [35] finding that patients with CTCs > 5/7.5 mL of blood have a lower OS. In this study, the authors also found that the number of CTCs in stage III GC patients was higher than that in stage I GC patients and that the overall survival rate of patients with more than 5 CTCs was lower. Dan Zeng et al. [37] used ligand‐targeted polymerase chain reaction to detect the levels of folate receptor-positive CTCs in blood samples from GC patients and found that preoperative CTCs levels have a diagnostic value in predicting peritoneal metastases in GC. Furthermore, Huang et al. [30] found CTCs in 10.8% of the resected group with a high recurrence rate and 60.2% of the unresectable group, but not in the healthy control group. After meta-analysis, they discovered that the incidence of CTCs in stage I/II GC was lower than in stage III/IV GC. In addition, Matsusaka et al. [34] and Yu et al. [38] identified CTCs in patients with progressive GC treated with neoadjuvant chemotherapy and surgery, and the OS and progression-free survival (PFS) were significantly shorter in patients treated with GC chemotherapy with a high number of CTCs. Therefore, all the preceding studies suggest that evaluating CTCs may be useful for predicting tumor progression and prognosis in GC patients. Monitoring the dynamics of CTCs in response to therapy may be a useful alternative method for assessing treatment resistance in GC patients.

Immune checkpoint blockers have rapidly gained popularity in the clinic as a novel antitumor treatment strategy in recent years [13]. Among these, programmed cell death protein 1 (PD-L1) has been extensively studied in tumor progression and metastasis and has received considerable attention [41, 42]. Meanwhile, the analysis of PD-L1 expression levels in CTCs is becoming more popular in oncology (including GC) [43, 44]. For example, Liu et al. [31] found that cell-surface vimentin (CSV) + PD-L1 + CTCs in patients with GC are associated with advanced disease and adverse effects. Cells with PD-L1 overexpression in the CSV + CTC cell population have a worse prognosis. Similarly, Cheng et al. [32] used CanPatrol CTCs enrichment technology on blood samples from 32 GC patients and found that the number and type of CTCs and CTCs-PD-L1 correlate with the clinical outcome of checkpoint blockade therapy. This evidence supports CTCs-PD-L1 expression as a prognostic factor for the efficacy of immune checkpoint blockade therapy.

#### Detection methods of CTCs

**Fig. 2:**
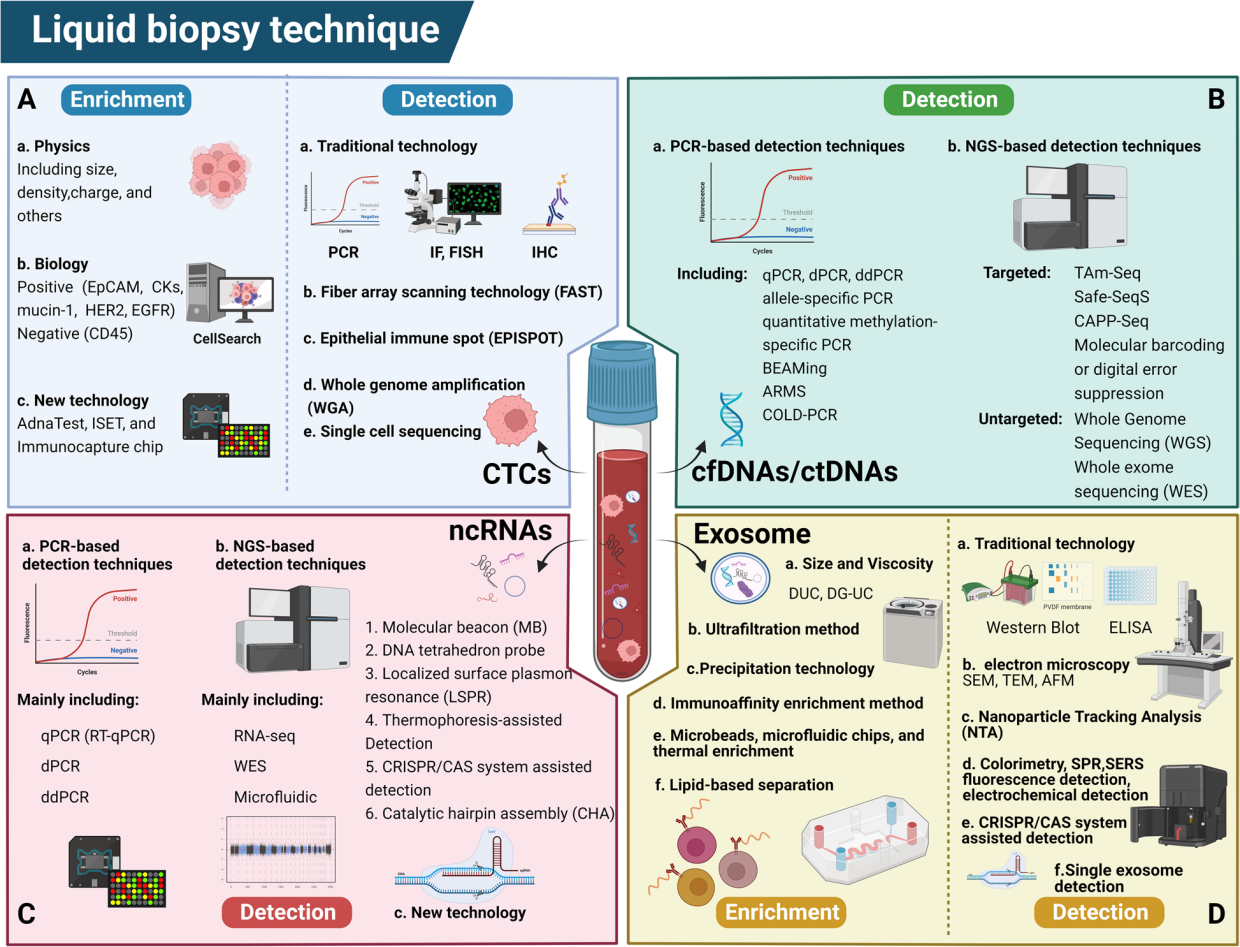
Techniques for detection of liquid biopsy biomarkers in gastric cancer (GC). Detection of CTCs (A), cfDNA/ctDNA (B), ncRNAs (C), and exosomes (D) in body fluids using different techniques can help in early diagnosis, prognosis prediction, treatment, and recurrence monitoring, and targeted therapy of GC. Created with BioRender.com

Since CTCs are difficult to detect in blood [21, 22, 45], establishing standardized detection methods for CTCs and investigating innovative techniques would increase the sensitivity and accuracy of diagnosing early malignant tumors (Fig. 2A) [46]. CTCs detection consists of three steps: enrichment, detection, and analysis. CTCs enrichment techniques include physical and biological enrichment. Physical enrichment does not require immunological labeling of CTCs and solely depends on their physical properties (including size, density, charge, and other biological properties) [47–49]. Bioenrichment relies on immunological antibodies, allowing for the specific capture of CTCs. This approach includes positive selection with antibodies against tumor-associated antigens such as EpCAM, CKs, mucin-1, human epidermal growth factor receptor 2, or epithelial growth factor receptor [50–52] and negative selection with antibodies against the common leukocyte antigen CD45 [53]. The CellSearch system (Veridex) is the most widely used antibody-based isolation technique and the only one approved by the Food and Drug Administration (FDA) for the detection of CTCs in the blood of some tumor patients [54–56], which has a greater benefit as a diagnostic and prognostic indicator for patients with metastatic disease [34, 37]. However, imposing EpCAM bias on the enriched CTCs population is an obvious drawback of immunocapture methods, including CellSearch [57]. Therefore, numerous new methods have been developed, such as AdnaTest, isolation by size of epithelial tumor cells, density gradient, microfiltration, microflow, and size-determining immunocapture microarrays in recent years [58, 59]. Furthermore, recent technological advances have enabled the isolation and analysis of single intact CTCs [60, 61].

CTCs detection and analysis techniques include traditional polymerase chain reaction (PCR) and cellular protein detection methods such as immunofluorescence, immunohistochemistry, and fluorescence-assisted in situ hybridization. The latest methods, such as high-throughput fiber-optic array scanning technology and epithelial immunospotting, can screen CTCs and detect proteins secreted by CTCs [62–65]. The most widely used technique remains PCR, particularly quantitative real-time PCR, which can reduce false-positive results in the data by determining the “cut-off” value. We can count CTCs in the blood by detecting traditional markers of CTCs such as CKs, CEA, TWIST [33], KRAS [66], and PRRX1 [67] and non-coding RNAs, including microRNAs (miRNAs) and Piwi-interacting RNAs [68–70]. With the advancement of technology, sequencing and histological techniques, such as whole genome amplification, single-cell sequencing methods, and proteomics methods, are increasingly used to detect CTCs [71]. Nagrath et al. [72] developed the “CTCs chip,” a microfluidic-based device for detecting CTCs with a significantly higher yield and purity. Using this innovative technology, CTCs can be captured, stained, and scanned directly from small volumes of blood. Several studies have demonstrated the efficacy of this method [73–77]. Significant advances in the technology for detecting CTCs in various tumors have opened more opportunities for diagnosing and treating GC.

### cfDNA / ctDNA

Mandel et al. [78] discovered DNA fragments in blood in 1948 and coined the term cfDNA. Thierry et al. [79, 80] used cfDNA to discover specific mutations and genetic changes in tumors in 1994. Since then, cfDNAs have received increasing attention. ctDNA is a subset of cfDNA derived from tumor cells [81, 82]. ctDNA is a small gene fragment derived from primary tumors, metastatic tumors, or even CTCs released into the bloodstream [83]. Most cfDNA fragments are 160–200 base pairs long, whereas ctDNA fragments are much shorter; both are double-stranded fragments [84, 85] with a short half-life (approximately 2 h) [86–88]. According to subsequent research, tumor load, tumor status, DNA elimination, degradation mechanisms, the inflammatory response, or tissue damage can affect the amount and nature of ctDNA [89, 90], allowing it to reflect tumorigenesis and progression in vivo in real time. Furthermore, ctDNA carries tumor-specific genetic/epigenetic variants (including point mutations, structural variants, copy number variants, microsatellite alterations, and methylation) that vary greatly between individuals [91], making ctDNA collection from blood non-invasive compared to traditional tumor biopsy and facilitating the identification and screening of GC. The clinical application of cfDNA/ctDNA as liquid biopsy markers in GC is listed in Table 2.

**Table 2:** Clinical application of cfDNA/ctDNA in GC

Object	Sample Type	Sample size	Sensitivity (%)	Specificity (%)	AUC	Clinical significance	References
cfDNA	Plasma	30 patients with GC	96.67	94.11	0.991	For early detection of cancer and assessment of tumor load	[92]
cfDNA	Serum	124 patients with GC	78.96	91.81	0.94	For early screening of GC	[93]
cfDNA	Serum/ Plasma	130 patients with GC	75	63	0.784	Plasma cfDNA levels are lower than serum cfDNA levels, and plasma cfDNA levels may help predict GC patients	[94]
cpDNA	Plasma	277 patients with progressive GC	34	^a^	^a^	Associated with poor prognosis in GC	[95]
ccf^a^DNA	Plasma	73 patients with GC	^a^	^a^	0.744	Distinguish between patients with stage III and IV GC and healthy controls, and monitor postoperative	[96]
cfDNA	Plasma	30 patients with locally progressive, unresectable, or metastatic GC	^a^	^a^	^a^	Positive correlation between tDNA levels and DFS in patients with progressive GC receiving systemic chemotherapy 3 months after the start of chemotherapy	[97]
ctDNA	Plasma	46 patients with stage I-III GC	39	100	^a^	MRD with ctDNA testing identifies patients at high risk of recurrence	[98]
cfDNA	Serum	428 patients with GC	68.9	95.8	0.98	Predicts response to chemotherapy and surgery in patients with colorectal cancer; tumor recurrence should be considered in GC with persistently elevated cfDNA levels after surgery	[99]
cfDNA	Plasma	106 patients with progressive GC treated with chemotherapy	93.7	45.2	0.8099	Tumor biomarkers as monitoring the efficacy of chemotherapy for GC	[100]
ctDNA	Plasma	61 cases of partially metastatic GC	^a^	^a^	^a^	Associated with improved prognosis	[101]
cfDNA	Plasma	1145 patients with GC	87	^a^	0.984	Potential to expand access to targeted therapies and immunotherapy to all patients with advanced cancer	[102]
Methylation of cfDNA	Plasma	89 patients with GC	40–60	92	0.89	Effective differentiation between early GC, colorectal cancer, and liver cancer	[103]
Methylation of cfDNA	Plasma	99 patients with GC	^a^	^a^	0.81	High postoperative long fragment LINE-1 concentrations suggest high risk of MRD and recurrence	[104]
Methylation of RASSF1A, SOX17 and WiF-1	Plasma	70 patients with progressive GC	^a^	^a^	^a^	Associated with worse progression-free survival and overall survival	[105]
Methylation of cfDNA	Plasma	1781 Gastrointestinal tumors	^a^	^a^	0.9	Improved diagnostic accuracy of gastrointestinal cancers	[106]

^a^indicates that this data was not presented in the study

#### Clinical application of cfDNA/ctDNA in GC liquid biopsy

Increasing evidence suggests that cfDNA/ctDNA is a new potential biomarker that can guide diagnosis, predict prognosis, monitor recurrence, and treat GC [107, 108]. In evaluating the potential therapeutic utility of plasma ctDNA levels as a diagnostic tool for early GC, Kim et al. [92] found that the area under the curve (AUC) value of cfDNA diagnostic GC was 0.991, and its sensitivity and specificity reached 96.67% and 94.11%. They also found that the level of cfDNA expression increased proportionally among healthy participants, patients with early GC, and patients with late GC, and that was associated with tumor node metastasis (TNM) staging. Furthermore, cfDNA levels are significantly reduced 24 h after surgery compared to pre-GC levels. Qian et al. [93] found that the level of serum cfDNA expression in patients with stage I GC was about 6 times that of the healthy control participants, and the level of cfDNA expression in patients with stage III to IV GC was significantly higher than that in patients with stage I GC. The AUC for diagnosing GC with cfDNA was 0.94, with sensitivity and specificity of 78.96% and 91.81%, respectively. Similarly, Park et al. [94] found that the average concentration of plasma cfDNA in patients with GC was 2.4 times that of the normal control participants, and the sensitivity and specificity of using cfDNA to distinguish between patients with GC and normal people reached 75% and 63%, respectively. Fang et al. [95] and Yang et al. [98] found that ctDNA positivity was related to the disease stage, that ctDNA could hardly be detected in early GC patients before surgery, that patients with high ctDNA levels have a significantly lower OS and a higher risk of peritoneal recurrence, and that ctDNA mutations are associated with a poor prognosis in patients with advanced GC. Similarly, Pu et al. [96] demonstrated that ctDNA could distinguish patients with advanced GC from patients with early GC and healthy controls, with AUC values of 0.565 and 0.744, respectively. Interestingly, they also found that ctDNA concentrations are high up to 21 days postoperatively but reduce after 3 months. In a separate investigation, Normando et al. [97] found significantly prolonged disease-free survival in patients with low ctDNA levels in GC after one cycle of chemotherapy. According to Lan et al. [99] cfDNA levels are more sensitive than CEA levels for predicting recurrence in surgically followed patients. Zhong et al. [100] found that plasma cfDNA concentrations tend to increase with GC progression by analyzing changes in plasma cfDNA concentrations during the chemotherapy treatment of patients with advanced GC. There is no significant difference in the trend of plasma cfDNA concentrations over time in patients with stable disease. The findings of the above studies indicate that cfDNA levels can be used to monitor the progression of GC.

As an essential component of liquid biopsy, cfDNA/ctDNA is also used in immuno-oncology. Numerous studies have demonstrated the efficacy of PD-1 targeted therapy for some patients with metastatic GC (MGC). To investigate the potential role of cfDNA/ctDNA in GC-targeted therapy, Kim et al. [101] conducted a prospective analysis of 61 MGC patients treated with pembrolizumab and observed ctDNA levels drop after 6 weeks of patient treatment. Moreover, microsatellite instability (MSI) predicted the ICB therapeutic benefit of PD-1/PD-L1 inhibitors [109]. Willis et al. [102] detected MSI in GC using a targeted next-generation sequencing (NGS) approach. They reported that using cfDNA, they could accurately detect 87% of tissue MSI positivity and 99.5% of tissue microsatellite stability, with an overall accuracy of 98.4%. In addition, immunotherapy is clinically active in cfDNA-MSI-positive patients with advanced GC, with more than half achieving complete or partial remission and sustaining therapeutic benefits. However, research on immune-targeted therapy for cfDNA/ctDNA and GC remains in its preliminary stages.

Aberrant DNA methylation is an epigenetic alteration occurring in organ disease specificity. Methylation of promoter regions has been widely used to identify cfDNA/ctDNA in GC plasma and serum, and frequent promoter hypermethylation and subsequent loss of protein expression are associated with GC [15]. Thus, detecting the cfDNA/ctDNA methylation signature has become an early diagnostic, prognostic prediction, and screening marker for GC. Ren et al. [103] developed a methylation CpG tandem amplification and sequencing (MCTA-Seq) and identified 153 cfDNA methylation biomarkers, including DOCK10, CABIN1, and KCNQ5, and their work showed that MCTA-Seq could distinguish early GC using a highly specific algorithm. MCTA-Seq detected early-stage GC at a sensitivity of 40%–60% with a specificity of 92%. While Kandimalla et al. [106] developed EpiPanGI Dx, a sensitive and targeted methylation-based assay for cfDNA that increases the prediction accuracy of large log GC to 85–95%. In a separate study, Ko et al. [104] found that LINE-1 methylation in cfDNA may serve as a novel biomarker for screening GC, with an AUC of 0.81 for its diagnostic GC. The findings also indicated that patients with lower pre-treatment LINE-1 methylation levels have significantly lower OS, and patients with low preoperative LINE-1 methylation have worse recurrence-free survival and OS. Karamitrousis et al. [105] used methylation-specific PCR to detect the methylation status of oncogenes *RASSF1A*, *SOX17*, and *WiF-1* in the cfDNA of 70 patients with progressive GC and found that promoter methylation of the examined genes is significantly associated with decreased PFS and OS compared with patients without methylation and that simultaneous methylation of the above genes results in worse PFS and OS in GC patients. Given the above findings, changes in cfDNA/ctDNA concentrations may be a reliable biomarker for detecting early GC.

#### Detection methods of cfDNA/ctDNA

Like CTCs, cfDNA/ctDNA levels are low in vivo and have a short half-life, necessitating sensitive and specific detection methods [110], which can be divided primarily into PCR-based and NGS-based methods (Fig. 2B). PCR remains the most widely used method among these. PCR-based techniques include standard quantitative PCR (qPCR), digital PCR (dPCR) and droplet digital PCR (ddPCR), allele-specific PCR, quantitative methylation-specific PCR, BEAMing (beads, emulsions, amplification, and magnetism) [111–113], ARMS [114], and co-amplification at lower denaturation temperature-PCR [115]. The sensitivity of PCR-based assays has increased dramatically over the past decade. On the other hand, dPCR and ddPCR techniques allow absolute quantification of target molecules, gradually allowing this technique to replace traditional detection methods. Although PCR-based techniques are typically more sensitive and less expensive, numerous experiments have shown that the number of mutations detected is limited, the detection area is somewhat restricted, and the sample size increases as the assay is used more frequently. This has increased the use of NGS technology for cfDNA/ctDNA detection, which can detect multiple mutations and new mutations.

NGS-based assays are categorized as targeted and untargeted, with targeted NGS technologies such as tagged-amplicon deep sequencing (TAm-Seq) [116], Safe-Sequencing System (Safe-SeqS) [117], and personalized profiling by deep sequencing (CAPP-Seq) [118], detecting multiple rare mutations in the genome and ctDNA simultaneously without tumor sequencing. Molecular barcoding or digital error suppression are additional techniques to differentiate between actual low-frequency mutations and artifactual mutations that appear during PCR amplification [119, 120]. Although targeted ctDNA analysis can identify tumor mutations in some patients, it does not rule out the possibility of patients developing novel, unknown mutations. Non-targeted NGS technologies, such as whole genome sequencing (WGS) and whole exome sequencing, can be used to detect all tumor mutations in patients and can also be used for whole genome copy number analysis and large structural variant detection, despite their low sensitivity and prohibitive cost [121].

Due to their respective advantages and disadvantages, there is currently no uniform standard for cfDNA/ctDNA detection assays. Combining the two approaches can increase the number and detection limits of cfDNA/ctDNA using WGS to map tumor-specific chromosomes in tumor tissues, followed by quantitative analysis of cfDNA/ctDNA in plasma using PCR- or NGS-based quantitative methods [122, 123]. However, cfDNA/ctDNA assays have limitations, and there is a need for sensitive, high-target volume, and low-cost assays.

### ncRNAs

In addition to the NGS-based genome sequencing described above, various transcriptome sequencing technologies have emerged, resulting in many coding RNAs/messenger RNAs (mRNAs) and ncRNAs. Although ncRNAs cannot be translated, they can act as “regulators” of many genes or proteins. Among the major ncRNAs, miRNAs, tRNA-derived small RNAs (tsRNAs), long non-coding RNAs (lncRNAs), and circular RNAs (circRNAs) have received the most attention [124]. miRNAs are small non-coding RNAs (sncRNAs) of about 22 nucleotides in length that can affect tumor biological progression by regulating tumor proto-oncogenes or oncogenes at the post-transcriptional level [125, 126]. Similarly, shorter-length tsRNAs are derived fragments generated after the cleavage of pre-tRNAs or mature tRNAs in specific environments [127, 128], which can be classified into tRNA-derived fragments and tRNA halves depending on the location of cleavage [129].

Furthermore, tsRNAs have more modifications than other sncRNAs, making them more stable in the blood. The mechanism of action of tsRNAs is similar to that of miRNAs [130, 131], and they play a role in tumor progression by regulating gene expression, translation, and epigenetics [132]. Unlike the sncRNAs mentioned above, lncRNAs are ncRNAs of more than 200 nucleotides in length, most of which are by-products of RNA polymerase II transcription and can play a key role in tumor progression through various mechanisms such as regulating target gene expression, recruiting chromatin modifications, interfering with mRNA splicing, translation, and degradation, and acting as miRNA sponges [124, 133–135]. circRNAs are covalently closed single-stranded cyclic molecules whose structure resists degradation by most ribonucleases R, making their expression more stable [136]. circRNAs can influence tumor biology by acting as “miRNAs sponges” or translation templates for certain peptides and proteins and binding to specific RNA-binding proteins [137–139]. Because of these ncRNAs’ unique structure and properties, their expression in blood is relatively stable, allowing them to serve as important biomarkers and therapeutic targets for many tumor liquid biopsies, including GC [107, 132, 140, 141]. The clinical application of ncRNAs as liquid biopsy markers in GC is listed in Table 3.

**Table 3:** Clinical application of ncRNAs and exosomes in GC

Projects	Category	Biomarker	Sample Type	Expression	Clinical significance	References
**Diagnosis, prognosis, and treatment**	miRNAs	miR-21、miR-106、miR-421 and miR-223	Serum, plasma, gastric juice, and blood	All were significantly different	Helps in early diagnosis and mass screening of GC	[142]
		miR-875-5p	Tissue and cells	downregulated	May serve as a potential diagnostic and therapeutic target for GC	[143]
		miR-148a	Plasma	downregulated	Associated with tumor progression and poor prognosis. Restoration of blood miR-148A levels may be a new nucleic acid anti-cancer therapy	[144]
		miR-23b	Plasma	up regulated	Associated with poor clinical prognosis	[145]
	circRNAs	circPTPN22	Plasma	up regulated	Can effectively identify GC patients and healthy controls	[146]
		hsa_circ_0045602、hsa_circ_0008768、hsa_circ_0007380、hsa_circ_0002019、hsa_circ_0006089、hsa_circ_0034398、hsa_circ_0052001 and hsa_circ_0001013	Plasma	up regulated	Powerful identification of early GC patients	[147]
	tsRNAs	hsa_tsr016141	Tissue and serum	up regulated	Clearly distinguish GC patients from healthy controls or patients with gastritis	[148]
	Exosome	Exosome miR-4741、miR-32、miR-3149 and miR-6727	Tissue and plasma	Expression of exosome miR-4741 was upregulated, and expression of exosome miR-32, miR-3149 and miR-6727 was downregulated	Act as a diagnostic marker for GC and an influential factor in inhibiting GC progression	[149]
		Exosome LncRNAH19GC	Serum	downregulated	A potential biomarker with diagnostic and prognostic value	[150]
		Exosome hsa_circ_0015286	Tissue, plasma, and cells	up regulated	May be a non-invasive biomarker for GC diagnosis and prognostic assessment	[151]
**Transfers**	miRNAs	miR-137-3p	Cells	downregulated	Tumor-suppressing effect, may promote GC progression by affecting immune infiltration	[152]
		miR-548d-3p	Cells	up regulated	Accelerated proliferation and migration of GC cells	[153]
		miR-4742-5p	Cells	up regulated	Effective treatment to inhibit cancer metastasis	[154]
		miR-17-5p	Cells	up regulated	Influence the transfer and progression of GC	[155]
	lncRNAs	PTCSC3	Tissue and cells	downregulated	Regulation of cell invasion and migration	[156]
	circRNAs	CircPVT1	Cells	up regulated	Regulation of GC cell proliferation, migration, and invasion	[157]
		circ_0006089	Cells	up regulated	Regulation of GC cell proliferation, migration, and invasion	[158]
	tsRNAs	tiRNA-Val-CAC-001	Tissue and cells	downregulated	As a promising biomarker and by targeting LRP6, regulates the Wnt/β-catenin signaling pathway to affect GC cell metastasis	[159]
	Exosome	Exosome TRIM3	Serum	downregulated	Inhibition of GC growth and metastasis in vitro and in vivo	[160]
		Exosome MET	Cells	up regulated	Promotes tumor growth and progression in vitro and in vivo	[161]
**Drug resistance**	miRNAs	miR100、miR-34a、miR-23a and miR-30a	_	All were significantly different	Modulated sensitivity of GC to chemotherapeutic agents	[162]
		let-7 g、miR-342、miR-16、miR-181、miR-1 and miR-34	_	All were significantly different	Correlation with chemotherapy sensitivity	[163]
		miR-30a	Cells	up regulated	Led to Beclin 1-mediated autophagy and promoted cisplatin-induced apoptosis and G2/M phase cell cycle arrest in GC cells	[164]
	lncRNAs	CRNDE	Cells	downregulated	Playing a key role in autophagy-mediated chemoresistance through binding to SRSF6	[165]
		SNHG6	Cells	up regulated	Progression of cisplatin resistance and GC is regulated by sponge miR-1297	[166]
	circRNAs	Hsacirc_004413	Cells	up regulated	GC cells can be made resistant to 5-fluorouracil by adsorption of miR-145-5p	[167]
		circ_AKT3	Tissue and cells	up regulated	Interaction with miR-206 regulates cisplatin resistance	[168]

#### Clinical application of ncRNAs in GC liquid biopsy

Tumor cells can deliver ncRNAs into body fluids via special mechanisms known as circulating ncRNAs. They are widely present in body fluids such as plasma, serum, and tumor patients’ exocytosis. Moreover, increasing studies have shown that circulating ncRNAs in the blood of tumor patients can be detected in large quantities and used as biomarkers for GC [169, 170]. Our previous studies have shown that circPTPN22, which is up-regulated in GC plasma, can effectively differentiate GC patients from healthy controls; its AUC value can reach 0.857. In addition, the level of expression of circPTPN22 was higher in patients with advanced GC (stages III-IV) than in patients with early GC (stages I-II). The expression level of circPTPN22 in n patients with GC decreased significantly following surgery, and its high expression predicted the existence of GC. The high expression of circPTPN22 indicated poor survival for patients with GC. This suggests that circPTPN22 can be used as an early diagnostic and prognostic marker for GC [146]. Meanwhile, another study also found that hsa_tsr016141, which was up-regulated in GC serum, could also effectively distinguish GC patients from healthy controls, its AUC value can reach 0.814 [148]. Recently, Roy et al. [147] developed a risk diagnosis prediction model based on 8 CircRNAs that are up-regulated in both tissues and plasma of GC patients. This model can accurately and effectively distinguish GC patients from non-disease control groups, and the AUC values of GC diagnosed by this model in the training cohort and testing cohort are 0.87 and 0.83, respectively. It is worth noting that this model can effectively identify early (stages I-II) GC patients in the training cohort and the validation cohort, and their AUC values can reach 0.87 and 0.82 respectively. In a recent meta-analysis, Xu et al. [142] found that miR-21, miR-106, miR-421, and miR-223 have better diagnostic efficacy for GC, with miR-421 particularly useful as an auxiliary diagnostic indicator for GC; the AUC of its diagnostic GC can reach 0.92. More studies found that miR-548d-3p [153], miR-4742-5p [154], and miR-23b [145], which are significantly up-regulated in GC, and miR-137-3p [152], miR-148a [144], and miR-875-5p [143], which are significantly down-regulated in GC, are effective in distinguishing GC patients from healthy control individuals. In terms of metastasis, miR-17-5p and miR-4742-5p are up-regulated in GC. Meanwhile, the knockdown of either miR-17-5p or miR-4742-5p significantly inhibits GC cell proliferation, invasion, and metastasis [154, 155]. Furthermore, down-regulated PTCSC3 expression in GC patients correlates with HULC, which is up-regulated in GC patients. PTCSC3 inhibits GC cell invasion and migration, whereas HULC promotes it. Both act on cell invasion and migration via the Wnt/β-catenin signaling pathway [156]. Another study found that knocking down the expression of up-regulated CircPVT1, or circ 0,006,089, in GC cells reduces GC cell growth, invasion, and migration [157, 158]. Zheng et al. [159] found that the tiRNA-Val-CAC-001, which is down-regulated in expression in GC tissues and cells, may affect GC cell metastasis by targeting LRP6 and regulating the Wnt/β-catenin signaling pathway. All the above studies suggest that ncRNAs play a crucial role in the early diagnosis, prognosis prediction, and metastasis of GC.

In addition to the early diagnosis and prognostic significance of GC, ncRNAs have been linked to susceptibility and resistance to anti-treatment in GC. Azimi et al. [162] and Kim et al. [163] used data mining and high-throughput miRNA microarray analysis to identify numerous miRNAs (miR100, miR-34a, miR-23a, miR-30a, let-7 g, miR-342, miR-16, miR-181, miR-1, and miR-34) associated with chemotherapy sensitivity in GC. Zhang et al. found that CRNDE, which is lowly expressed in human GC, may play an important role in autophagy-mediated chemoresistance by binding to SRSF6. When GC patients develop chemoresistance, CRNDE expression could be restored to improve the effect of chemotherapy, implying that CRNDE may be a new biomarker for GC prognosis and treatment [165]. In addition, ncRNAs have gained interest as GC-targeted drugs for treating GC. Cisplatin (CDDP) chemotherapy significantly reduces the level of miR-30a in GC cells, and interestingly, up-regulation of miR-30a inhibits GC cell sensitivity to CDDP [164]. Furthermore, CDDP-resistant GC cells that up-regulated SNHG6 and circ_AKT3 expression could regulate CDDP resistance and GC progression via sponge miR-1297 and miR-206, respectively, indicating that targeting SNHG6 and circ_AKT3 may be a promising option to address GC chemoresistance [166, 168]. In 5-fluorouracil (5-FU)-resistant GC cells, hsacirc_004413 could make GC cells resistant to 5-FU by adsorbing miR-145-5p [167]. circCPM up-regulates another 5-FU-resistant GC cell line and tissues and increases PRKAA2 expression by directly binding to miR-21-3p, activating GC cell autophagy and chemoresistance [171]. ncRNAs have been considered biomarkers for multiple-stage cancers. If ncRNA assays can be used effectively in clinical practice, these methods can detect tumors in patients early and mitigate their suffering [172].

#### Detection methods of ncRNAs

Because ncRNAs and cfDNA are nucleic acid products, they are detected similarly. RT-qPCR, dPCR, and ddPCR based on PCR reactions, gene chips, and NGS-based RNA-sequencing are the most common methods for detecting ncRNAs (Fig. 2C). RT-qPCR is the gold standard method for quantitatively detecting trace ncRNAs, with high sensitivity, reproducibility, and accuracy [173]. dPCR and ddPCR aid in quantifying ncRNAs and allow for the precise quantification of target nucleic acids in samples. For initial screening and obtaining profiles of ncRNAs, gene microarray and NGS methods are commonly used [174], whereas RT-qPCR and dPCR are used to validate previous results [175].

Chen et al. [176] recently proposed RNAdetect, a computational method that incorporates novel predictive features based on generalized ensemble defects. Furthermore, n-gram models extract features that effectively capture sequence homology with known ncRNA families. Novel methods for detecting ncRNAs have emerged as technological advancements. Catalytic hairpin assembly (CHA), molecular beacon, DNA tetrahedron probe, localized surface plasmon resonance, thermophoresis-assisted detection, and CRISPR/CAS system-assisted detection [177–180] are just a few examples. Bellassai et al. [181] developed a hairpin-probe-based isothermal strand replacement polymerization method for detecting miRNAs and used it to quantify osteoarthritis-associated miR-127 in joint fluid. Furthermore, Chen et al. [182] coupled an RNA-based CHA circuit with CRISPR-Cas12a for detecting miRNAs under isothermal conditions. As a result, the circuit they developed achieved the nanomolar detection limit and allowed accurate detection of miRNA levels in different cell lines. Similarly, Yang et al. [183] proposed an isothermal amplification system based on double CHA and chameleon DNA template silver nanoclusters for label-free ratio detection of circRNAs. This system can detect and visually distinguish circRNAs. Its lower limit of detection for target circRNAs can reach 1 pm. Introducing these new technologies revitalizes the detection line of ncRNAs in blood and provides a new reference for further improving liquid biopsy rules.

### Exosomes

Exosomes are lipid bilayer extracellular vesicles with a diameter of 30–150 nm found in nearly all body fluids (blood, urine, cerebrospinal fluid, and saliva) [160, 184]. Exosomes secrete various mRNAs, ncRNAs, transmembrane or encapsulated cytoplasmic proteins, and lipids from tumors or normal cells into body fluids, allowing intercellular communication or releasing contents to facilitate fluid biopsy [185–189]. Because of the lipid bilayer structure, exosomes are relatively abundant and stable in circulation, making them potential biomarkers for early diagnosis, prognosis, treatment efficacy, and drug resistance in GC [190, 191]. The clinical application of exosomes as liquid biopsy markers in GC is listed in Table 3.

#### Clinical application of exosomes in GC liquid biopsies

Exosomes’ properties make them a promising candidate for liquid biopsy [185, 192]. First, blood exosomes contain many ncRNAs as biomarkers for early diagnosis, prognosis prediction, metastasis, and drug resistance in GC. Tang et al. [149] used precipitation technology to separate and extract exosomes from the plasma of 60 patients with early GC, 60 patients with intermediate and late GC, and 57 healthy people who served as controls. After analysis, the expression of exosome miR-4741 was up-regulated in GC tissues and plasma, while exosomes miR-32, miR-3149, and miR-6727 were down-regulated in GC tissues or plasma. The expression levels of miR-4741 and miR-3149 in the plasma of patients with early and middle-late GC were significantly different. The AUC values of the secretions miR-4741, miR-32, miR-3149, and miR-6727 for GC diagnosis were 0.8554, 0.9456, 0.7683, and 0.8923, respectively, suggesting that the above four secretions miRNAs can be used as GC diagnostic markers. Another study found that the expression level of exosomal LncRNAH19 in the serum of patients before and after GC surgery is significantly elevated, but the serum level of exosomal LncRNAH19 is significantly lower in patients after GC surgery compared to before surgery, the AUC of its diagnostic GC can reach 0.849, the sensitivity and specificity are 74.36% and 83.95%, respectively, and its expression level is significantly correlated with TNM stage [150]. Zheng et al. [151] detected that the up-regulation of hsa_circ_0015286 in GC tissue, plasma, and cancer cell exosomes is closely correlated with tumor size, clinical stage, and lymph node metastasis and that the AUC of GC was 0.778, with sensitivity and specificity of 82.1% and 65.7%, respectively. After surgery, the expression level of exosomal hsa_circ_0015286 is significantly lower in GC patients, and the OS time is significantly longer in patients with low exosomal hsa_circ_0015286 expression.

In addition to ncRNAs, exosomes contain cell-specific proteins that play an important role in GC diagnosis, prognosis, metastasis, and drug resistance. TRIM3 protein levels in serum exosomes of GC patients are significantly lower than those of healthy control individuals. Exosomes-mediated delivery of TRIM3 protein inhibits GC growth and metastasis in vitro and in vivo, suggesting that TRIM3 could be used as a diagnostic marker and therapeutic target for GC [193]. Furthermore, exosomes released by *Helicobacter pylori*-infected GC cells increase MET expression. These exosomes containing MET protein are delivered and internalized by macrophages, promoting tumor growth and progression in vitro and in vivo [161].

Taken together, circulating exosomes and their derived “products” may open new avenues for GC liquid biopsy.

#### Detection techniques of exosomes

Exosomes are difficult to isolate with high efficiency and purity in liquid biopsies due to their unique formation and delivery processes [194, 195]. Furthermore, because tumor exosomes constitute only a small proportion of all exosomes in body fluids, high-sensitivity and specificity exosome detection methods are needed for subsequent detection and analysis. Several methods have been developed for isolating exosomes and detecting exosomal proteins and nucleic acids (Fig. 2D) [196–198]. Exosome isolation and enrichment methods are based on exosome characteristics such as density, size, surface composition, and exosome precipitation [194, 199]. 1. Ultracentrifugation (UC) is based on particle size and solution viscosity and includes differential UC and density gradient UC [200]. UC is the gold standard for exosome isolation and the most widely used method [201]. 2. Ultrafiltration is a simple method with high purification but low yield based on exosome size [202]. Combination UC and ultrafiltration methods are now widely used, combining the advantages of both methods and making exosome extraction easier and yielding more exosomes. 3. The precipitation-based exosome separation and enrichment method allows exosome separation using highly hydrophilic polymers competitively bound to water molecules around the exosome membrane, reducing solubility [203]. 4. The immunoaffinity enrichment method for exosome isolation allows good differentiation between cancer cell-originated exosomes and normal cell-originated exosomes using antibodies and inducers targeting tumor-associated proteins such as CD81, GPC-1, and EpCAM [204, 205]. 5. The separation and enrichment of exosomes were mediated by lipid bimolecular affinity. This method can substantially shorten the separation procedure for exosomes [206]. 6. The use of microbeads, microfluidic chips, and thermal enrichment makes the enrichment of exosomes fast and simple [207–209].

The traditional method for detecting exosomes is using western blot analysis or an enzyme-linked immunosorbent assay for exosomal membrane proteins or other marker proteins, but the method is complicated, insensitive, and unsuitable for mass detection [210]. Therefore, new techniques for detecting exosomes have been developed, such as scanning electron microscopy, transmission electron microscopy, atomic force microscopy, dynamic light scattering, nanoparticle tracking analysis, colorimetric assay, fluorescence detection, surface plasmon resonance, surface-enhanced Raman scattering, magnetic detection, electrochemical detection, CRISPR/CAS system-assisted detection, single exosome detection, and others [211–218]. In the CRISPR/Cas9 system, Cas9 nuclease can recognize specific complementary double-stranded DNA (dsDNA) sequences containing proto-spacer adjacent motifs using guide RNA to efficiently shear dsDNA sequences. Protein detection can be transformed into nucleic acid quantification by targeting extracellular protein aptamers [219]. Recently, Zhao et al. [220] proposed an aptamer-based extracellular membrane protein recognition combined with CRISPR/Cas12-assisted fluorescent signal amplification, making the CRISPR/Cas system promising as a sensitive tool for exosomal protein identification and quantification, which may aid in cancer diagnosis and therapeutic monitoring. Endogenous heterogeneity currently impedes the analysis of humoral exosomes, and in vivo mutations may lead to erroneous analysis results. Single exosome detection may offer a solution to this problem [221]. Guo et al. [218] used DNA nanostructure labeling and exosome membrane staining to establish a single-strand DNA-assisted single exon detection platform. The authors used long, single-stranded DNA amplified by the roll ring grown in situ on the surface of the exosome to identify the surface protein of a single exosome. This method can also analyze the protein mass spectra of different exosomes without calibration, allowing exosomes to be clearly distinguished based on their source cells.

## Challenges and future developments in liquid biopsy

Although liquid biopsy technology has promising applications, numerous issues must be addressed before clinical promotion. Table 4 summarizes the advantages and disadvantages of liquid biopsy techniques in GC.

**Table 4:** Liquid biopsy technique in GC

Testing Target	Testing purpose	Testing Technology	Testing Advantages	Testing Disadvantages	References
CTCs	Isolation and enrichment	Micro-electro-mechanical system (MEMS)	Simple and easy to operate	The large difference in size of different tumor cells, etc., makes it impossible to uniformly	[47]
		Isolation by size of epithelial tumor cells(ISET)			[48]
		Microfluidics			[49]
		Dual-Aptamer-Targeted Immunomagnetic Nanoparticles	Dependence on immunological antibodies for specific capture of CTCs	Recovery of EpCAM + CTCs only	[50]
		CellSearch	Semi-automatic and can process multiple samples at once	Enriched CTCs population exerts EpCAM bias	[37]
	Testing	qPCR and protein detection techniques (immunofluorescence, immunohistochemistry, fluorescence-assisted in situ hybridization.)	Enables quantitative and accurate testing	Longer detection time and less specificity	[33, 66, 68]
		Fiber-optic array scanning technology (FAST)	Detect low levels of CTCs without enrichment	Higher cost	[63]
		Epithelial immune spots	Detection of proteins secreted/released/shed by individual epithelial cancer cells	Smaller sample size for one-time processing	[64]
		Whole genome amplification (WGA)	Rapid and efficient detection of specific proteins or markers of CTCs	Lower specificity and higher cost	[71]
		Single-cell sequencing	Ability to detect its properties at the individual cell level	Lower level reflected	[71]
cfDNA/ctDNA	PCR-based detection	qPCR、dPCR、ddPCR、allele-specific PCR, quantitative methylation-specific PCR, BEAMing, ARMS, and COLD-PCR	Quantitative detection of target molecules with improved detection sensitivity and lower cost	The number of mutations detected is limited, the detection area is restricted, and the sample size increases with the number of times the assay is used	[111–115]
	NGS-based detection	Targeted NGS Technology(TAm-Seq, Safe-SeqS, CAPP-Seq and molecular barcoding or digital error suppression)	Can identify tumor mutations in some patients with high sensitivity	The number of detected tumor mutations is low and contains a large number of positional tumor mutations that are not detected	[116–120]
		Non-targeted NGS technology(WGS and WES)	Detects all tumor mutations in patients and can be used for genome-wide copy number analysis and large structural variant detection	Lower sensitivity and higher cost	[121]
ncRNAs	PCR-based detection	RT-qPCR, dPCR, and ddPCR	Better sensitivity, repeatability, and accuracy	The number of molecules detected is limited and the sample size increases with the number of times the assay is used	[174]
	NGS-based detection	Gene chips	Preliminary screening and mapping of ncRNAs can be obtained	Lower specificity and higher cost	[175]
		RNA-Seq	Preliminary screening and mapping of ncRNAs can be obtained	Lower specificity and higher cost	[175]
	Detecting the presence of functional ncRNAs	RNAdetect	Accurate detection of the presence of functional ncRNAs	High dependency	[176]
	Isothermal amplification technique detection	Catalytic hairpin assembly (CHA)	The detection conditions are relatively simple and allow for amplification of the target molecule, making it easy to detect	Poor specificity	[183]
	Detection of new technologies	Molecular beacons (MB), DNA tetrahedron probe, Localized surface plasmon resonance (LSPR), Thermophoresis-assisted detection and CRISPR/CAS system-assisted detection	Easy and convenient operation, lower detection limit	Poor specificity or low sensitivity	[177–180]
Exosome	Isolation and enrichment	Differential Ultracentrifugation (DUC) and Density Gradient Ultracentrifugation (DG-UC)	Exosome isolation and enrichment based on particle size and solution viscosity, simple operation and high extraction purity	Lower recovery efficiency and purity	[201]
		Ultrafiltration	Simple operation, high purification	Lower yield	[202]
		Sedimentation Technology	Highly hydrophilic polymers can be used to competitively bind to water molecules around the exosome membrane, thereby reducing solubility and enabling exosome separation	Lack of specificity, serious contamination, difficult to expand	[203]
		Immunoaffinity enrichment method	High specificity, better differentiation between exosomes of cancer cell origin and exosomes of normal cell origin	Higher cost and high dependency	[205]
		Microbeads, microfluidic chips, and thermal enrichment	Improved efficiency and detection limits of exosome enrichment	Higher cost and high dependency	[207–209]
		Lipid‑based separation	Reduce the damage of exosome and extract effectively	With other pollution and lack of specificity	[206]
	Testing	Traditional Western Blot (WB) or enzyme-linked immunosorbent assay (ELISA)	Specifically detects exosomal membrane proteins or other marker proteins	Complex steps and low sensitivity	[210]
		Scanning electron microscopy (SEM), transmission electron microscopy (TEM), atomic force microscopy (AFM), and dynamic light scattering (DLS)	Easy, fast, and visual operation	Samples are susceptible to damage from external conditions and are more expensive	[211]
		Nanoparticle Tracking Analysis (NTA)	Visualize and provide numerous information on exosomes	Complex operation and poor repeatability	[160]
		Colorimetric method	Easy to operate and visualize	High dependent and subject to external interference	[215]
		Fluorescence detection	Low cost, non-destructive, multiplex detection capability, easy automation	High dependent and subject to external interference	[213]
		Surface Plasmon Resonance (SPR)	No sample preparation and a label-free, real-time molecular sensing technique	High dependent	[214]
		Surface enhanced Raman scattering (SERC)	Enabled cheap, portable, and easily available establishment of detection	Higher cost and high dependency	[217]
		Electrochemical testing	High sensitivity, high selectivity, low cost, easy to use	High dependent	[212]
		CRISPR/CAS system-assisted detection	High specificity	Complex operation and low sensitivity	[187, 213, 215]
		Single exosome detection	Provide more accurate tumor progression information and reflect individual differences, and have strong specificity	Low detection rate and high personalization cost	[218]

First, a highly sensitive assay must be developed. For example, CTCs and cfDNA/ctDNA concentrations in GC patients’ blood are low, but the sensitivity of current detection and extraction techniques for both is low, making detection more challenging [222, 223]. Furthermore, although NGS technology can improve detection sensitivity through enrichment and amplification, it also introduces issues such as gene information mismatches, incomplete detection information, and false positives [224]. Furthermore, most current technologies have long detection times and high costs, limiting their use to large-scale promotion. Therefore, technologies with high sensitivity and high detection efficiency are required to support liquid biopsy.

Second, standardized operational procedures and data processing methods must be developed. Since existing clinical guidelines for liquid biopsy do not provide uniform and prudent evidence, the liquid biopsy results are not very comparable, and the assay quality is not uniform. In addition, the sensitivity and specificity may differ when using different techniques or assays to detect markers [15]. Therefore, before liquid biopsy can be used in clinical practice as a precision medicine tool to drive GC management, pre-analytical procedures, and post-analytical data processing, such as counting CTCs and ctDNA, characterization of CTCs and genetic or epigenetic changes in ctDNA analysis, and quantification of circulating ncRNAs, must be standardized to ensure reproducibility and data comparability.

Third, large-scale clinical study validation is an important aspect of the clinical application of liquid biopsy technology. Current liquid biopsy marker studies include fewer samples and have a short validation period. In addition, most of them focus only on the specificity and sensitivity of the liquid biopsy detection system without a comprehensive test of the assay’s reproducibility, accuracy, consistency, reference range, and minimum detection limit. For example, CTCs and cfDNA studies have primarily focused on the late-stage or postoperative blood of tumor patients, with few studies on their different stages. Studies on cfDNAs, ctDNAs, and ncRNAs, on the other hand, have focused on detecting the sensitivity and specificity of markers in different stages or states of tumors, with few studies exploring the scope of their detection. In contrast, histology studies are more pre-screened, which results in more markers being obtained at the end, lowering the final specificity. These issues require large-scale clinical samples for subsequent demonstration and provide sufficient evidence and support for liquid biopsy technology’s early entry into clinical applications.

Fourth, novel biomarker types are being investigated as liquid biopsy reserves. There are currently fewer types of liquid biopsies, and the relatively small amounts of CTCs, ctDNAs, ncRNAs, and exosomes included compared to other components of blood make detection more difficult [225]. Therefore, researchers have also developed more content-rich biomarkers to complement them. Currently, tumor-educated platelets, circulating endothelial cells, and tumor microenvironment components have been identified as liquid biomarkers for biopsy [226–228], but no application of these three markers in GC diagnosis has been found. Therefore, more research is needed to complement the effective GC markers and find new markers that can compensate for the shortcomings of current biomarkers and thus provide clinical guidance.

Finally, the future of liquid biopsy will be a combined diagnosis. Although many liquid biopsy techniques have been developed and many biomarkers have been screened using various techniques, studies have shown that combining different biomarkers for a tumor or disease diagnosis and drug use improves overall accuracy. For example, Li et al. [229] found that using tRNA-GlyGCC-5 and sRESE alone has a much lower effect on identifying esophageal squamous cell carcinoma than using both. Hong et al. [230] and Tang et al. [231] found that the combination of exosome miRNAs or the combination of exosome miRNAs and CEA surpassed a single exosome miRNA marker in the diagnosis of GC. Our previous research found that the AUC value of GC diagnosis using circPTPN22 alone was 0.857, but the AUC value increased to 0.892 after combining traditional tumor markers CEA and CA199, demonstrating that the combined use of markers could compensate for the limitations of a single marker [146]. Therefore, if the benefits of various liquid biopsy techniques can be fully utilized and the combination of these techniques can be used for tumor diagnosis, it will significantly impact the future diagnosis and treatment of tumors and diseases.

## Conclusion

In conclusion, as a non-invasive detection method, liquid biopsy has emerged as a promising new modality for GC in early screening or diagnosis, postoperative monitoring, treatment response, and tumor drug resistance [232]. The method provides tumor molecular information and overcomes tumor heterogeneity, allowing real-time monitoring of tumor progression and personalized patient treatment. Although liquid biopsy has demonstrated significant benefits, the lack of novel liquid biopsy markers, low specificity and sensitivity, a lack of standard operating procedures and data analysis methods, and prohibitive costs currently prevent liquid biopsy from being widely used in the clinic. We believe that as the mechanisms of GC development and detection technology advance, the role of liquid biopsy technology in GC will gradually be revealed. It will be of great value in the clinical application of GC diagnosis and prognosis.

## Data Availability

Not applicable.

## Abbreviations

GC: Gastric cancer
CEA: Carcinoembryonic antigen
CA: Carbohydrate antigen
CTCs: Circulating tumor cells
cfDNA: Circulating free DNA
ctDNA: Circulating tumor DNA
ncRNAs: Non-coding RNAs
EpCAM: Epithelial cell adhesion molecules
CKs: Cytokeratins
OS: Overall survival
PFS: Progression-free survival
PD-L1: Programmed cell death protein 1
CSV: Cell-surface vimentin
FDA: Food and Drug Administration
PCR: Polymerase chain reaction
miRNAs: MicroRNAs
TNM: Tumor node metastasis
MGC: Metastatic GC
MSI: Microsatellite instability
NGS: Next-generation sequencing
qPCR: Quantitative PCR
dPCR: Digital PCR
ddPCR: Droplet digital PCR
TAm-Seq: Tagged-amplicon deep sequencing
Safe-SeqS: Safe-Sequencing System
CAPP-Seq: Personalized profiling by deep sequencing
WGS: Whole genome sequencing
mRNAs: Messenger RNAs
tsRNAs: TRNA-derived small RNAs
lncRNAs: Long non-coding RNAs
circRNAs: Circular RNAs
sncRNAs: Small non-coding RNAs
AUC: Area under the curve
CDDP: Cisplatin
5-FU: 5-Fluorouracil
CHA: Catalytic hairpin assembly
UC: Ultracentrifugation
dsDNA: Double-stranded DNA
